# In vivo wear performance of highly cross-linked polyethylene vs. yttria stabilized zirconia and alumina stabilized zirconia at a mean seven-year follow-up

**DOI:** 10.1186/1471-2474-14-154

**Published:** 2013-05-01

**Authors:** Masahiro Hasegawa, Akihiro Sudo

**Affiliations:** 1 Department of Orthopaedic Surgery, Mie University Graduate School of Medicine, Tsu City Mie 514-8507, Japan

**Keywords:** Total hip arthroplasty, Zirconia, Highly cross-linked polyethylene, Wear, Transformation rate

## Abstract

**Background:**

Zirconia was introduced as an alternative to alumina for use in the femoral head. The yttria stabilized zirconia material was improved by adding alumina. We evaluated highly cross-linked polyethylene wear performance of zirconia in total hip arthroplasty. The hypothesis was that alumina stabilized zirconia could decrease highly cross-linked polyethylene wear.

**Methods:**

Highly cross-linked polyethylene wear was measured with a computerized method (PolyWare) in 91 hips. The steady-state wear rates were measured based on the radiographs from the first year postoperatively to the final follow-up and were compared between hips with yttria stabilized zirconia and alumina stabilized zirconia.

**Results:**

The steady-state wear rate of highly cross-linked polyethylene against zirconia was 0.02 mm/year at a mean follow-up of 7 years. No significant difference was observed between groups with yttria stabilized zirconia and alumina stabilized zirconia.

**Conclusions:**

Addition of alumina to the zirconia material failed to show further reduction of highly cross-linked polyethylene wear and our hypothesis was not verified.

## Background

Polyethylene wear debris is a known cause of osteolysis and loosening in total hip arthroplasty (THA). Femoral heads with improved surface characteristics have been introduced to reduce the wear. In an attempt to reduce polyethylene wear, ceramic femoral heads have been used as an alternative bearing surface. Alumina ceramic was first introduced as a ceramic material for femoral heads. Alumina ceramic has excellent geometric form and wettable surface, enhances hardness, thereby maintaining lubrication, and increases resistance to third-body wear [1]. Despite the excellent wear characteristics of alumina ceramic, a historic problem has been that, under certain conditions, it is prone to fracture [2].

Zirconia ceramic was introduced in 1985 as an alternative to alumina ceramic for the femoral head. Zirconia is tougher and more resistant to fracture [3,4]. Zirconia ceramic has three phases of physical structure. The tetragonal phase has the greatest toughness and is used for the manufacture of prosthetic femoral heads, but it is also the most unstable phase with possible surface transformation back into the more stable monoclinic phase [5,6]. Alloying pure zirconia with stabilizing oxides such as MgO and Y_2_O_3_ allows the retention of the tetragonal structure at room temperature [7]. Yttria-stabilized tetragonal zirconia polycrystal (Y-TZP) has been used extensively. However, with Y-TZP, the potential exists for late phase transformation and aging, resulting in grain pullout, surface cracking, and increasing surface roughness [8]. In one study of 52 retrieved Y-TZP heads, the monoclinic content within the bearing surface averaged 40% compared with 3% in nonimplanted heads [8,9]. The tetragonal to monoclinic phase transformation of zirconia can be suppressed by the addition of a small amount of aluminum oxide (Al_2_O_3_). Recent improvements achieved by the addition of 0.25 wt% of Al_2_O_3_ have increased the static and dynamic fracture strengths of zirconia, giving higher resistance to low-temperature aging degradation and phase transformation [10]. On the other hand, highly cross-linked polyethylene has been shown to achieve wear reduction compared with conventional polyethylene using a hip simulator [11]. Several clinical studies have also shown improved wear rates for highly cross-linked polyethylene cups compared with conventional polyethylene cups against cobalt-chromium heads in midterm follow-up [12-14]. If ceramic heads and highly cross-linked polyethylene cups were coupled, further reductions in polyethylene wear could be expected. A few studies examined the clinical efficacy of this combination of highly cross-linked polyethylene cup with a zirconia head [13,15-17]. However, no reports have yet examined the influence of improvement of the material for polyethylene wear. The purpose of this study was to evaluate the clinical outcomes and *in vivo* polyethylene wear performance of yttrium-oxide-partially-stabilized zirconia (PSZ) and alumina stabilized zirconia (ASZ) after a minimum 5-year follow-up. Both zirconia (PSZ and ASZ) were manufactured by Japan Medical Materials Corp. (JMM, Osaka, Japan). The hypothesis was that ASZ could decrease wear of highly cross-linked polyethylene. Retrieved PSZ and ASZ heads from this cohort of patients were also compared to never-implanted heads.

## Methods

From January 2001 to September 2004, we performed 117 primary cementless THA with a zirconia head in 100 consecutive patients. These patients were 88 women and 12 men with a mean age of 60 years (range, 21–85 years) and a mean body mass index of 23.3 kg/m^2^ (range, 16.0-33.7 kg/m^2^). The preoperative diagnoses were osteoarthritis including congenital dislocation in 75 patients (90 hips), idiopathic osteonecrosis of the hip in 14 patients (15 hips), and rheumatoid arthritis in 11 patients (12 hips). All operations were performed by a single surgeon (A.S.) via a posterior approach. We excluded hips followed for less than 5 years from this study (Table 1).

**Table 1:** Demographic characteristics of two groups

	**All**	**PSZ**	**ASZ**	**P value**
Number of Hips	91	23	68	
Gender (men/women)	8/83	2/21	6/62	>0.999
Age (years)	59	64	57	0.02
Body Mass Index (kg/m2)	23.4	24.1	23.2	0.32
Diagnosis				
Osteoarthritis	75	16	58	0.21
ION	10	5	6	
Rheumatoid Arthritis	6	2	4	
Polyethylene Thickness (mm)	11	10.7	11.5	0.020

*ION* idiopathic osteonecrosis of the hip.

*PSZ* partially-stabilized zirconia.

*ASZ* alumina stabilized zirconia.

The cementless cup consisted of a titanium alloy metal shell (QPOC; JMM, Osaka, Japan) and a highly cross-linked ultra–high-molecular-weight polyethylene shell (Excellink; JMM, Osaka, Japan). The surface of the metal shell was porous-coated with pure titanium and a coating of apatite-wollastonite (AW) glass-ceramic was applied to the porous area. The metal shell was fixed with titanium screws to ensure primary fixation. The highly cross-linked polyethylene shell was gamma-irradiated to 50 kGy, heat annealed, and sterilized with 25 kGy of gamma radiation in nitrogen. The stem was a HS-6 collarless stem made of titanium alloy and was proximally porous-coated with an AW glass-ceramic. The head was a 26-mm yttria-stabilized zirconia head secured with a Morse taper. Improvements have been achieved by the addition of 0.25 wt% of Al_2_O_3_ (ASZ) since 2002.

Four patients (5 hips) died from unrelated medical conditions within 5 years after the THA and one patient was lost to follow-up. Three patients (3 hips) underwent revision due to infection (1 hip) and recurrent dislocation (2 hips) within 5 years after the THA. Thirteen patents (17 hips) underwent clinical evaluation without radiographs in our institution after a minimum follow-up of 5 years. After excluding those patients, 79 patients (91 hips) underwent both clinical and radiographic evaluations after a minimum follow-up of 5 years (mean, 7 years; range, 5–9.2 years). Sixty-eight hips received an improved zirconia head. Demographic characteristics of two groups are shown in Table 1. Full weight bearing was allowed 5 days postoperatively. Clinical and radiographic evaluations were scheduled immediately, after 6 weeks, 3 months, 6 months, and 1 year, and then annually after surgery. This study was approved by the institutional review board of Mie University Hospital, and all patients gave informed consent for the report to be published.

Clinical evaluation was performed using the Merle d’Aubigné and Postel scoring system [18]. We assessed the score before primary arthroplasty and at the last follow-up. At each follow-up, standardized anteroposterior and lateral radiography of the hip was performed without weight-bearing. Anteroposterior radiographs were taken of bilateral hip joints with the patient in a supine position. Serial radiographs were analyzed by the same independent observer (M.H.). We used three radiographs per patient to determine the polyethylene wear. We selected the 3-week postoperative radiograph as a baseline. In addition, we analyzed the 1-year and last follow-up radiographs. We performed radiographic measurements of two-dimensional femoral head penetration into the polyethylene with a computerized method (PolyWare 5.10 digital version; Draftware Developers Inc., Vevay, IN) validated for clinical measurement of polyethylene wear in metal-backed acetabular components [19]. Linear wear was measured in the plane of the anteroposterior radiograph by the software with an accuracy of 0.022 mm [19,20]. Penetration of the femoral head into the acetabular polyethylene is due to a combination of creep and wear. The total head penetration rate was calculated based on the latest follow-up radiographs by dividing the observed head penetration by the postoperative period. Because creep deformity could be gained until about 1 year postoperatively [12,13], the steady-state wear rates were measured based on the radiographs from the first year postoperatively to the final follow-up. Radiographic loosening of the acetabular component was evaluated using the method of Hodgkinson et al. [21]. Radiographic loosening of the femoral component was evaluated using the method of Engh et al. [22]. The inclination of the cup was measured. We compared the polyethylene wear between PSZ and ASZ. The effects of several factors on wear rates were examined, including sex, age, body mass index, liner thickness, cup inclination, and postoperative Merle d’Aubigné and Postel score [18].

Three zirconia femoral heads, retrieved at the time of revision procedures due to infection and recurrent dislocation, were analyzed to determine the surface roughness (Ra) using a contact surface profilometer (SV-3100; Mitutoyo Corporation, Kawasaki, Japan). The parameter Ra was the mathematical average of all deviations (peaks and valleys) from the mean line of the surface profile. The monoclinic content of the surface of the zirconia head was calculated with x-ray diffraction (PW3050; Spectris Co. Ltd., Tokyo, Japan). Scanning electron microscopy was used to evaluate the topography of the retrieval surfaces. The durations of implantation of these three zirconia femoral heads were 1.5, 2.3, and 3.2 years. Two unused zirconia heads were also examined. One head did not contain alumina (PSZ) and the other head contained alumina (ASZ).

Statistical analysis was performed using the Wilcoxon signed rank test and the Mann–Whitney U test for continuous variables. The chi-squared test and Fisher’s exact test was used for categorical data. Correlation analysis was performed using Spearman’s rank correlation test. A multiple regression test was also used. Statistical significance was set at p < 0.05. Kaplan-Meier survivorship for the entire population of 117 hips was analyzed with revision as the endpoint in 100 patients.

## Results

### Clinical results

Osteolysis was not found around the implant and no zirconia head had a fracture. No hip showed findings of aseptic loosening; however, one hip showed acetabular loosening because of late infection. Postoperative dislocation occurred in five hips. Three patients (three hips) underwent revision from 1.5 to 3.2 years after the initial operation. Of these, one hip underwent a revision because of infection with acetabular loosening. In two patients, the components were revised because of recurrent dislocation. The clinical results of the 79 patients (91 hips) revealed that the mean Merle d’Aubigné and Postel score for this group had improved significantly from 9.6 points (range, 2 to 16) preoperatively to 15.3 (range, 7 to 18) (p < 0.001) postoperatively. The mean scores for pain, mobility, and walking ability improved from 2.8 (range, 0 to 5), 4.4 (range, 0 to 6), and 2.3 (range, 0 to 6) preoperatively to 5.6 (range, 3 to 6), 5.5 (range, 2 to 6), and 4.3 (range, 0 to 6) at the last follow-up, respectively.

### Polyethylene wear

**Figure 1:**
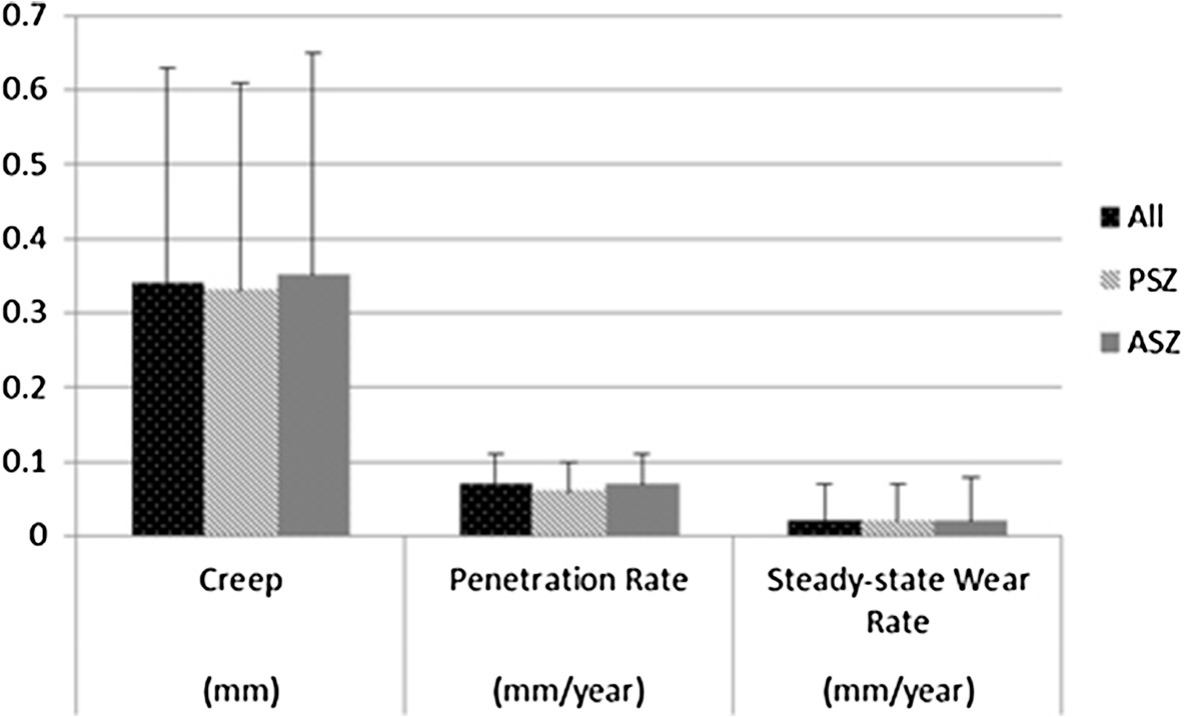
Creep, penetration rate, and steady-state wear rate of highly cross-linked polyethylene (mean ± standard deviation).

The linear wear of polyethylene against a zirconia head, including creep and steady-state wear, is shown in Figure 1. The mean steady-state wear rate was very low (0.02 mm/year). No significant difference was observed between PSZ and ASZ (creep, p = 0.590; penetration rate, p = 0.066; steady-state wear, p = 0.725). Steady-state wear was not associated with sex (p = 0.844). No significant correlations were observed between steady-state wear and age (R = −0.055, p = 0.600), body mass index (R = −0.138, p = 0.191), liner thickness (R = 0.092, p = 0.383), cup inclination (R = 0.021, p = 0.839), and postoperative Merle d’Aubigné and Postel score (R = 0.091, p =0.387). After adjusting polyethylene wear of the zirconia material for age and polyethylene thickness, polyethylene wear showed no differences between PSZ and ASZ (creep, R^2^ = 0.049, p = 0.111; penetration rate, R^2^ = 0.048, p = 0.117; steady-state wear, R^2^ = 0.012, p = 0.596). Kaplan-Meier survivorship with an endpoint of revision was 97% (95% confidence interval [CI], 94% to 100%) at 7 years.

### Retrieval study

**Figure 2:**
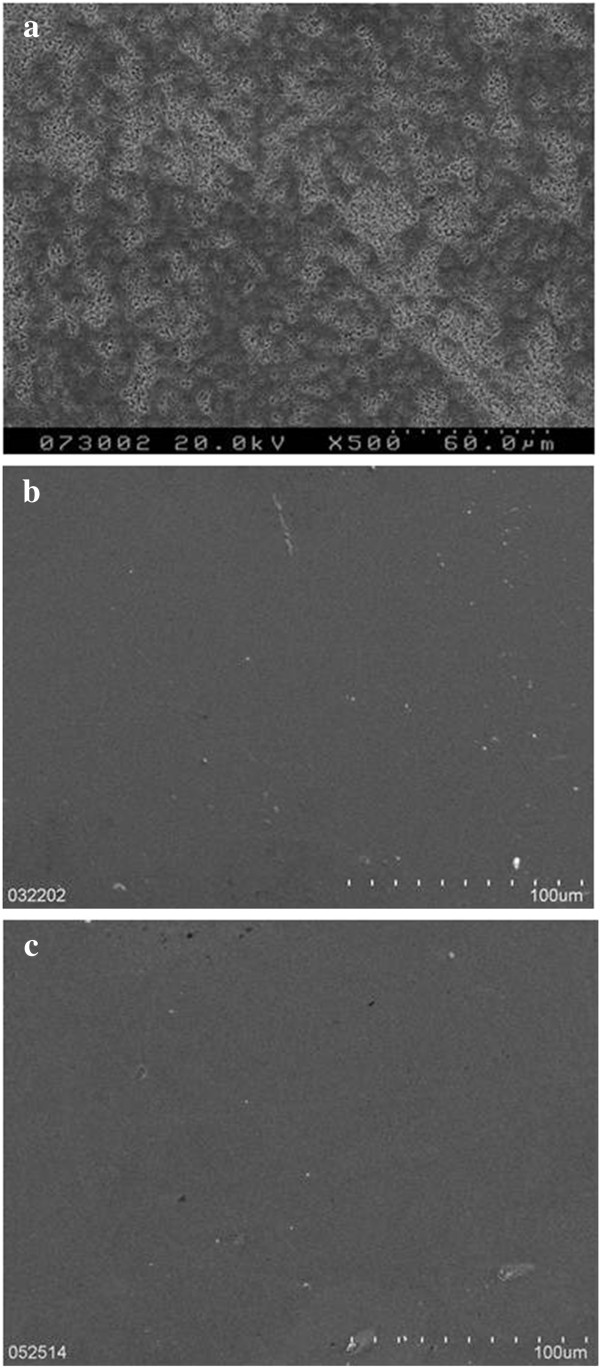
**The photomicrographs showing the topography of retrieval heads at the pole region: (a) PSZ (aged 2.3 years in vivo), (b) ASZ (aged 1.5 years in vivo), and (c) ASZ (aged 3.2 years in vivo).** PSZ head had a bumpy surface, whereas ASZ heads showed no apparent roughening.

The transformation rates from the tetragonal phase to the monoclinic phase as well as the Ra values of the three retrieved zirconia heads and two unused heads are shown in Table 2. One retrieved head with a transformation rate of 51% represented the material of PSZ. The rate dropped to 7% in two retrieved ASZ. Scanning electron microscopy revealed widespread irregularities on the surface of PSZ head and smooth surfaces of ASZ heads (Figure 2).

**Table 2:** Transformation rate and surface roughness of retrieved and unused zirconia heads

**Cause of Revision**	**Zirconia Material**	**Duration (years)**	**Transformation rate (%)**	**Surface roughness Ra (μm)**
Infection	PSZ	2.3	51	0.06
Dislocation	ASZ	1.5	7	0.03
	ASZ	3.2	7	<0.01
Unused	PSZ		4	0.01
	ASZ		3	<0.01

*PSZ* partially-stabilized zirconia.

*ASZ* alumina stabilized zirconia.

## Discussion

The addition of alumina to the zirconia material contributed to the reduced transformation rate; however, further reduction of highly cross-linked polyethylene wear was not achieved.

Previous retrieval studies reported that the average roughness of Y-TZP heads increased with age *in vivo*, and all but one also reported increased tetragonal-to-monoclinic phase transformation [23]. Such phase transformation can be accompanied by a 3% change in the volume of the ceramic head and may cause an increase in its surface roughness [4]. Fernandez-Fairen et al. [8] showed that the monoclinic content in the bearing surface of 47 retrieved heads ranged from 16% to 65%. In the present study, the transformation rate was 51% in heads without alumina; however, the rate decreased to 7% in heads containing alumina. Hayaishi et al. [24] demonstrated that transformation rate was 13% in Y-TZP heads containing 0.01% alumina (NGK Spark Plug, Nagoya, Japan). A laboratory study showed that Y-TZP heads revealed transformation from the tetragonal to monoclinic phase after 20 hours of autoclaving. The transformation rate was limited to 7-17% for heads containing alumina, whereas heads without alumina transformed at a higher rate, 58-78%, which is a fourfold difference [25]. Increased monoclinic content was associated with longer time from implantation. An increase in monoclinic content was correlated with a decrease in fracture toughness, increase in surface roughness, and increase in surface wear (Table 3) [8,23,24,26-28]. The dislocation and reduction maneuvers may roughen the head leading to inaccurate measurements of surface roughness. Gentle reduction is required. Kim et al. [29] showed a metal transfer on a ceramic femoral head correlated with increased surface roughness. In contrast, Haraguchi et al. [27] suggested that recurrent dislocation might not have been the cause of the surface roughening.

**Table 3:** Summary of maximum transformation rate and surface roughness of retrieved yttria stabilized zirconia heads

	**N**	**Duration**	**Transformation rate**	**Surface roughness**
		**(years)**	**(%)**	**Ra (μm)**
Hayaishi et al. [24]	1	1	13	0.01
Hernigou et al. [26]	3	8-11	30	0.05
Haraguchi et al. [27]	2	3, 6	37	0.12
Roy et al. [23]	7	0.04-11	49	0.03
Fernandez-Fairen et al. [8]	47	2-10	65	0.30
Santos et al. [28]	18	0.2-10	70	0.04
Present study	3	1.5-3	51	0.06

The theoretical reduction in polyethylene wear with the use of zirconia femoral heads is based on their high surface hardness, high compressive strength, high wettability, and superior surface polish [30]. Kim [4] reported the results of a prospective randomized study of 28-mm cobalt-chromium and Y-TZP femoral heads on conventional polyethylene. This is the only study to confirm a midterm wear analysis favoring zirconia-on-polyethylene (0.08 mm/year) versus cobalt-chromium-on-polyethylene (0.17 mm/year). Most reports showed no differences in terms of conventional polyethylene wear against between Y-TZP heads and cobalt-chromium heads (Table 4) [4,9,13,20,31]. The wear rate of polyethylene against Y-TZP heads was reported to be significantly greater than the wear rate against alumina heads (Table 4) [10,26]. Until 1998, the incidence of Y-TZP head fractures was 1 in 11,000 [32]. However, a report in the early 2000s demonstrated a fracture rate of 1 in 4 in certain batches [33]. This led to a recall of Y-TZP heads by the United States Food and Drug Administration in 2001 and suspension of production by the French authorities [32].

**Table 4:** Comparison of polyethylene wear against yttria stabilized zirconia, cobalt chromium, and alumina heads

	**Mean Followup**	**Penetration Rate (mm/year)**			**P value**
	**(years)**	**Yttria Stabilized Zirconia**	**Cobalt Chromium**	**Alumina**	
Stilling et al. [20]	5	0.23	0.25		0.46
Cohn et al. [9]	4	0.14	0.11		0.07
Kim [4]	7	0.08	0.17		0
Kraay et al. [31]	4	0.06	0.05		n.a.**
Nakahara et al. [13]	7	0.03*	0.03*		0.77
Hernigou et al. [26]	5	0.04		0.04	n.a.**
	12	0.41		0.07	0.01
Liang et al. [10]	5	0.13		0.08	<0.01

*highly cross-linked polyethylene.

** not available (no significance).

Recent clinical studies have reported a low wear rate of highly cross-linked polyethylene against zirconia (Table 5) [13,15-17]. However, no reduction was found in wear when using Y-TZP compared with cobalt-chromium femoral heads [13,15-17,23]. These results could be explained by the fact that the wear rates were nearly zero and it might be hard to measure the difference between Y-TZP and cobalt-chromium. Roy et al. explained that the failure to reduce wear might be due to phase transformation and degradation of Y-TZP femoral heads [23]. In contrast, magnesia-stabilized zirconia resists phase transformation and degradation *in vivo* and in artificial aging studies, maintaining its smooth surface finish and hardness [23].

**Table 5:** Summary of highly cross-linked polyethylene wear against yttria stabilized zirconia heads

	** Polyethylene**	**Zirconia**	**N**	**Mean followup**	**Steady-state wear rate**		**P value**
				**(years)**	**Yttria stabilized zirconia**	**Cobalt chromium**	
					**(mm/year)**		
Nakahara et al. [13]	Longevity (Zimmer)	(NGK)	51	7	−0.01	−0.01	0.83
Kawate et al. [16]	Aeonian (JMM)	(JMM)	32	5	0.00	0.01	0.36
Fukui et al. [17]	Longevity (Zimmer)	(NGK)	45	5	0.01		
Miyanishi et al. [15]	Aeonian (JMM)	(JMM)	95	2	0.06		
Present study	Excellink (JMM)	(JMM)	91	7	0.02		

The present study failed to show further reduction of highly cross-linked polyethylene wear using the new zirconia. The mean age of the patients with the new zirconia was 57 years as opposed to 64 years in the patients with PSZ. Since patients are usually more active when they are younger, it is not fair to conclude that the newer-style heads did not contribute to lower wear. Hernigou and Bahrami [26] showed that the increase in rate of conventional polyethylene wear against Y-TZP head was only evident after eight years and might be linked to a long-term biodegradation of zirconia in vivo. Comparing new head with old head in the early years, the wear may be similar, but as time goes on we might see a difference in this cohort. Long-term wear studies are warranted. This study has some limitations. First, the small number of retrieval heads was studied. Second, this study has no comparison with cobalt-chromium heads. Third, the mean apparent measured steady-state wear rates (0.02 mm/year) were actually lower than the accuracy threshold of our method (0.022 mm).

## Conclusions

The steady-state wear rate of highly cross-linked polyethylene against PSZ and ASZ heads were very low. No mechanical failures of the polyethylene or zirconia occurred. While our numbers were insufficient to compare degradation of material, addition of alumina to the zirconia material (ASZ) contributed to a reduction in the transformation rate; however, further reduction of highly cross-linked polyethylene wear was not achieved and our hypothesis was not verified.

## Competing interests

The authors declare that they have no competing interests.

## Authors’ contributions

MH and AS participated in the design and the coordination of the study. MH drafted the manuscript. Both authors read and approved the final manuscript.

## Pre-publication history

The pre-publication history for this paper can be accessed here:

http://www.biomedcentral.com/1471-2474/14/154/prepub
